# Spatial-Temporal Variation of Drought in China from 1982 to 2010 Based on a modified Temperature Vegetation Drought Index (mTVDI)

**DOI:** 10.1038/s41598-017-17810-3

**Published:** 2017-12-12

**Authors:** Shuhe Zhao, Dianmin Cong, Kexun He, Hong Yang, Zhihao Qin

**Affiliations:** 1 School of Geographic and Oceanographic Sciences, Nanjing University, 163 Xianlin Ave, Qixia District, Nanjing, 210023 China; 2 Jiangsu Provincial Key Laboratory of Geographic Information Science and Technology, Nanjing University, 163 Xianlin Ave, Qixia District, Nanjing, 210023 China; 3 Jiangsu Center for Collaborative Innovation in Geographical Information Resource Development and Application, Nanjing, 210023 China; 4 China Automotive Technology & Research Center, 68 East Xianfeng Road, Dongli District, Tianjin, 300300 China; 5 Norwegian Institute of Bioeconomy Research (NIBIO), Postboks 115, 1431 Ås, Norway; 6 CEES, Department of Biosciences, University of Oslo, Blindern, 0152 Oslo, Norway; 7 Institute of Agricultural Resources and Regional Planning, Chinese Academy of Agricultural Sciences, Beijing, 100081 China

## Abstract

Droughts cause huge losses of society and environment, therefore it is important to study the spatial-temporal pattern of drought. The traditional remote sensing drought indices (AVI, VCI and TCI) only consider the single factor representing the soil moisture (surface temperature or NDVI). The comprehensive remote sensing drought indices (VSWI and TVDI) can estimate the soil moisture more accurately, but they are not suitable for large scale region especially with great elevation variation. In this study, a modified Temperature Vegetation Drought Index (mTVDI) was constructed based on the correction of elevation and dry edge. Compared with the traditional drought indices, mTVDI had a better relationship with soil moisture in all selected months (R = −0.376, −0.406, −0.459, and −0.265, *p* < 0.05). mTVDI was used to analyze the spatial-temporal patterns of drought in China from 1982 to 2010. The results showed that droughts appeared more frequently in Northwest China and the southwest of Tibet while drought centers of North and Southwest China appeared in Huanghuaihai Plain and Yunnan-Guizhou Plateau respectively. The frequency of drought was increasing as a whole while the frequency of severe drought increased significantly by 4.86% and slight drought increased slowly during 1982 to 2010. The results are useful for the understanding of drought and policy making of climate change.

## Introduction

Droughts can cause great effects on the spatial-temporal pattern of water and heat in regional or even global scales^1^. Frequent severe droughts seriously reduce water supply and produce remarkable impact on ecosystem and social-economic development in the world^2–4^. China is located in East Asian the monsoon climate zone, where the climate and terrain are varied and complex. Global climate change has changed the spatial and temporal distribution of water and heat resources in China, thus leading to changes in biogeochemistry cycles^5,6^.

The seasonal variation of precipitation controlled by atmospheric circulation has been changed by climate change. Drought, the extreme shortage of rainfall, has been more and more affected by climate change^7,8^. For example, the droughts occurred in North China in 2009 caused 157 million acres of farmland being affected, and the severe droughts occurred in Southwest China from 2009 to 2010 made up to 120 million acres of breadbaskets production reduced^9^. Droughts have affected about 1/6 of China’s cultivated land each year^10^ and they caused a great loss of grain production. It is estimated that the crop yield reduction caused by drought is about 1500–2500 million tons per year, accounting for about 4–8% of grain production and more than 55% of total losses from natural disasters in China^7^. Thus, it is of great importance to research the spatial-temporal variation of drought in China for the sustainable development, particularly the agriculture and food security.

*In situ* measurement of soil moisture is the most accurate method for drought monitoring^11,12^. However, this method is constrained by high cost, manpower requirement, and limited spatial cover in big area, especially in remote areas with little access^12^. Thus, it is often used as proof data to validate the accuracy of other drought indices or to improve the spatial resolution of other data. There exist several drought indices based on meteorological data from meteorological stations, such as Standard Precipitation Index (SPI) and Reconnaissance Drought Index (RDI)^13^. But meteorological data encounters the similar problem of limited observation sites^12^. Remote sensing has the advantages of scanning the entire region, shortly revisiting to the same region, and conveniently accessing to the data. With the rapid development in the last decades, remote sensing technology has been widely used for drought monitoring, especially for the temporal-spatial evolution of drought^14–16^.

The Vegetation Index methods have been developed for the drought detection. Compared with the Normalized Difference Vegetation Index (NDVI), the Vegetation Condition Index (VCI) proposed by Kogan^17^ can describe the effect of weather on vegetation. However, the accuracy of the VCI is degraded by the time lag between precipitation and NDVI^14^. Then, the Anomaly Vegetation Index (AVI) was developed which was based on a normalization of NDVI values^18^. The Temperature Condition Index (TCI) was then developed by Kogan^19^ and it used the brightness temperature values to estimate drought by combining the VCI and TCI. Kogan^20^ and Unganai *et al*.^21^ proposed a new Vegetation Health Index (VHI). Rojas *et al*. used the VHI to assess drought in Africa and obtained relatively more accurate estimate of drought during the growth stage of crops^22^. Goetz^23^ and Carlson *et al*.^24^ found a significant negative relationship between the NDVI and temperatures (Ts).Carlson *et al*. proposed a new index called the Vegetation Supply Water Index (VSWI) based on the division of NDVI and LST^24^. Gillies *et al*.^25^, Carlson *et al*.^26^ and Price^27^ researched the relationship between NDVI and Ts and found that the NDVI-Ts space was a triangular shape when the vegetation cover was full. Based on the triangular shape, a comprehensive index, TVDI, was proposed by Sandholt *et al*.^28^ which had been used in several studies^29–31^. TVDI is comparatively empirical and is usually used in a small region. Kimura^32^ developed an improved temperature-vegetation dryness index (iTVDI) and it has been used to estimate the surface moisture condition in a river basin on the Loess Plateau, China. The iTVDI was used for soil moisture monitoring by incorporating air temperature in the TVDI equation^33^. However, these methods are all limited in the drought monitoring for large scales with great variation of elevation and complex climate.

Due to the vast territory and complex climate in China, the accuracies of drought measurement using indices leave much to be desired. Thus, it is very important to further improve the accuracy of the existing drought indices for the drought monitoring in China. In addition, the spatial-temporal pattern of droughts in China in the last decades is far from fully understood. In this paper, a modified Temperature Vegetation Drought Index (mTVDI), which is more suitable to monitor drought for the large scale region with complex climate and variable terrain, was developed based on the correction of elevation and dry edge of the existing Temperature Vegetation Drought Index (TVDI). The mTVDI was also used to estimate the spatial-temporal pattern of droughts in China during the period of 1982–2010. The aims of this research are: (1) to estimate the accuracy of the mTVDI by comparing it with traditional drought indices (AVI, VCI, TCI, VSWI and TVDI); (2) to estimate the spatial distribution and temporal variation of drought, particularly severe drought in China during 1982 to 2010.

## Results

### Validation of mTVDI based on *in situ* soil moisture data

**Figure 1:**
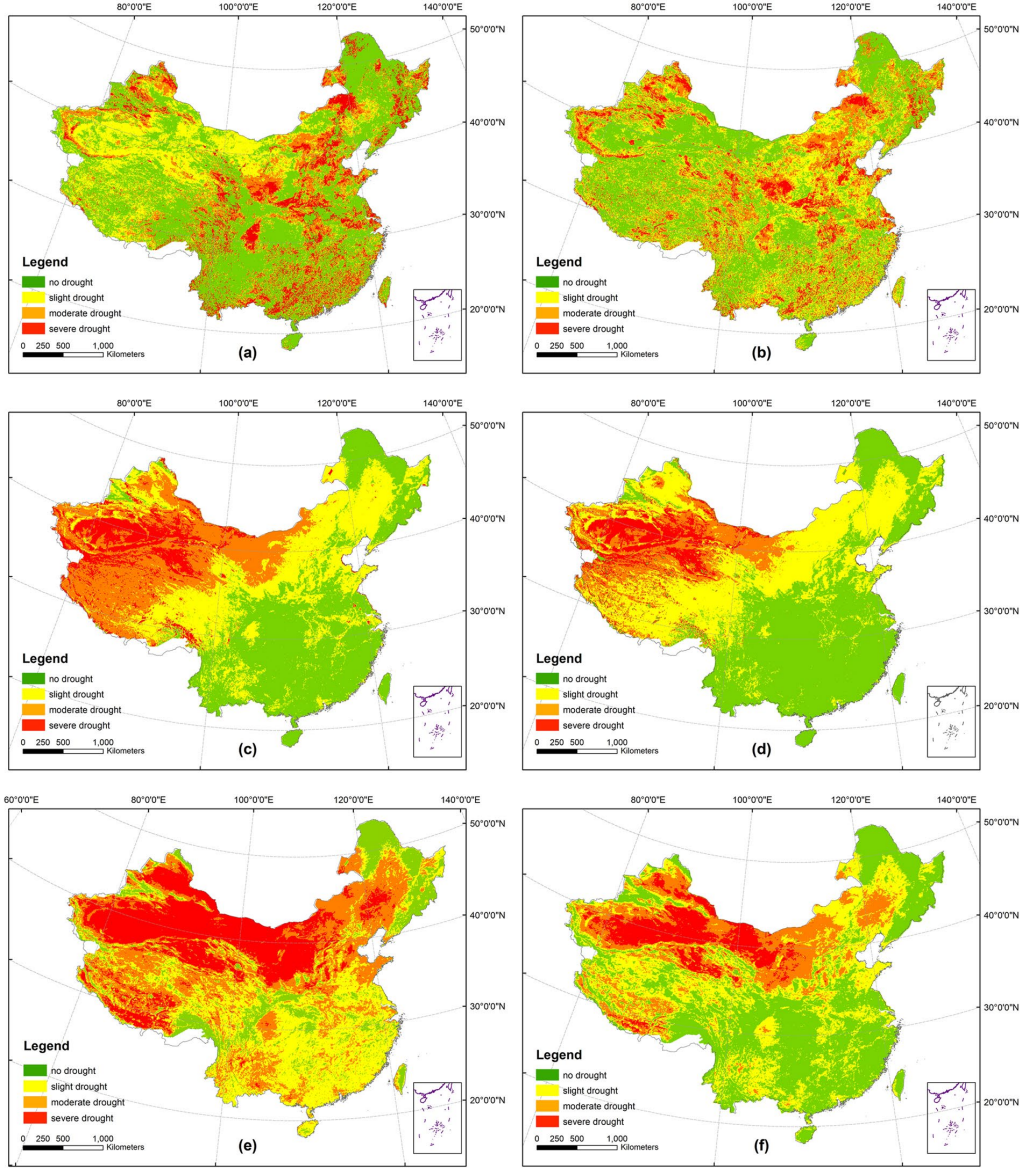
Comparison of drought distribution of the six drought indices in China in May 2007: (**a**) AVI, (**b**) VCI, (**c**) TCI (**d**) VSWI (**e**) TVDI and (**f**) mTVDI. The map was created using ArcGIS 10.2 (ESRI Inc., Redlands, California, USA).

The spatial distribution of the six drought indices (AVI, VCI, TCI, VSWI, TVDI and mTVDI) in May 2007 is shown in Fig. 1. AVI and VCI show discrete severe drought in some parts of the south of China, while the deserts in the northwest are no drought or slight drought, which obviously not coincide with the reality. TCI and VSWI show that the drought in China had become more and more serious from the southeast to the northwest, and the drought regions which belong to the same class are a centralizing and continuous distribution. But the drought in Xinjiang and Qinghai provinces are excessive. As for TVDI, it can accurately identify the position of drought regions in the northwest, but it seems show too much drought regions in the whole China. Compared with TVDI, the excessive drought in south China is corrected and the drought in western China is more apparent. Due to the elevation correction, uncertainty of drought estimate in the high altitude areas also could be further reduced.

To validate the sensitivity of mTVDI for soil moisture, this paper utilized the *in situ* soil moisture data to verify the mTVDI and traditional drought indices. Four months (March, May, July and November) for each season, which could present the drought and non-drought period during a year, in 2001, 2003, 2005, 2007, and 2009 are chosen to validate the reliability of the drought index mTVDI. And the statistical metrics of correlation coefficient (R), the relative bias (Bias), the root-mean-square error (RMSE) and the mean absolute error (MAE) were used (Table 1). As shown in Table 1, mTVDI had the highest R of −0.376, −0.406, −0.459, and −0.265 in the four selected months and AVI and VCI had the lowest R. Besides, the value of Bias, RMSE and MAE of mTVDI are relatively smaller than the other four indices, just in some cases bigger than that of TVDI. In conclusion, the mTVDI has the highest reliability in drought monitoring with a large scale among the six drought indices.

**Table 1:** Correlations results between drought monitoring indices and *in situ* soil moisture measurements.

Month	Index	R	Bias	RMSE	MAE
3	AVI	0.059	−0.384	0.340	0.297
	VCI	0.120	−0.339	0.415	0.342
	TCI	0.215	−0.635	0.504	0.456
	VSWI	−0.251	−0.584	0.504	0.449
	TVDI	−0.348	−0.082	0.277	0.217
	mTVDI	**−0.376**	−0.098	0.261	0.208
5	AVI	0.162	−0.331	0.291	0.246
	VCI	0.189	−0.301	0.391	0.323
	TCI	0.287	−0.485	0.387	0.337
	VSWI	−0.279	−0.659	0.512	0.460
	TVDI	−0.307	0.074	0.226	0.178
	mTVDI	**−0.406**	0.023	0.223	0.179
7	AVI	0.153	−0.308	0.289	0.246
	VCI	0.181	−0.318	0.408	0.330
	TCI	0.400	−0.254	0.277	0.227
	VSWI	0.408	−0.760	0.601	0.557
	TVDI	−0.183	0.001	0.210	0.169
	mTVDI	**−0.459**	−0.141	0.242	0.202
11	AVI	−0.056	−0.330	0.322	0.273
	VCI	−0.023	−0.447	0.475	0.397
	TCI	0.186	−0.612	0.509	0.474
	VSWI	−0.231	−0.688	0.569	0.534
	TVDI	−0.116	−0.102	0.203	0.167
	mTVDI	**−0.265**	−0.135	0.214	0.178

### Drought levels based on mTVDI

Drought is usually characterized by soil moisture (SM). According to the grade of meteorological drought from the National Standard of People’s Republic of China GB/T 20481-2006^34^, there is no drought when SM is greater than 60%; there is slight drought when SM is between 50% and 60%; there is moderate drought when SM is between 40% and 50%; and there is severe drought when SM is less than 40%. Thus, the linear relationship between mTVDI and SM is obtained based on the soil moisture data and the value of mTVDI, and their relationship is shown as Eq. (1).

1 $$y=-0.0024\ast x+0.8647$$

where $y$ represents the soil moisture; $x$ represents the mTVDI. According to the Eq. (1), there is no drought when mTVDI is less than 0.70, slight drought when mTVDI is between 0.70 and 0.75, moderate drought when mTDVI is between 0.75 and 0.80, and severe drought when mTVDI is more than 0.80.

### Spatial distribution of drought frequency based on mTVDI in China from 1982 to 2010

**Figure 2:**
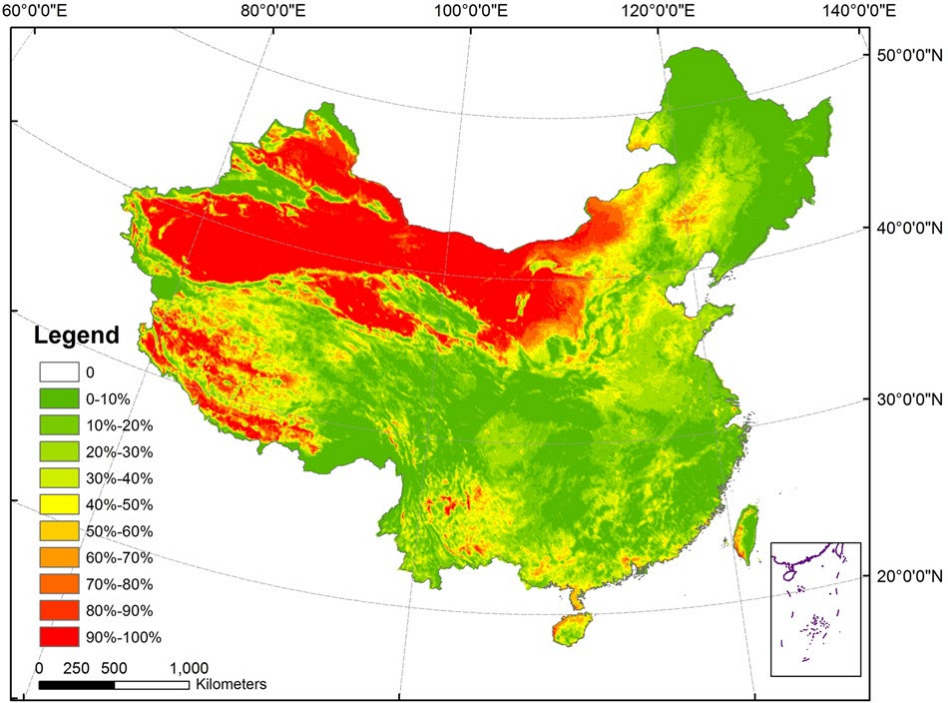
Spatial distribution of drought frequency in China from 1982 to 2010. The map was created using ArcGIS 10.2 (ESRI Inc., Redlands, California, USA).

This paper classified the drought into four levels: no drought, slight drought, moderate drought and severe drought based on the linear relationship between mTVDI and soil moisture in Eq. (1). To estimate the spatial distribution and temporal variation of the drought in China, times of different levels of drought (slight drought, moderate drought, and severe drought) were counted and the frequency of drought during the 324 months from 1982 to 2010 was calculated. Years of 1994 and 2000 were excluded due to the unavailable data. Drought frequency is defined as the number of drought events occurred^35^, but the drought frequency was expressed as percentage in this paper. The value not only could reflect the relative amount of drought occurred among all the pixels, but also we can see the proportion of drought occurred in a certain pixel during the long time period from 1982–2012. The spatial distribution of drought frequencies from 1982 to 2010 in China is shown in Fig. 2.

There are obvious spatial variations of drought frequency in China (Fig. 2). Due to the vast area of the desert, the drought frequencies in Northwest China were very high (>80%). Drought frequencies in the whole Yunnan-Guizhou plateau were relatively low, but drought frequencies in some areas in the plateau, particularly mountainous areas in Northwest Yunnan and Southwest Guizhou, were higher (>60%). The drought frequencies in most areas in Xinjiang were very high (>70%). The drought frequencies in Southwest Tibetan Plateau, the very high altitude area, were also very high. The drought frequencies in Huanghuaihai plain in North China were relative higher (40–60%). The drought frequencies in Southeast and Northeast China were relatively lower (<20%). The drought frequencies in the middle and lower reaches of the Yangtze River Plain and the eastern coastal areas were much lower (<20%).

**Figure 3:**
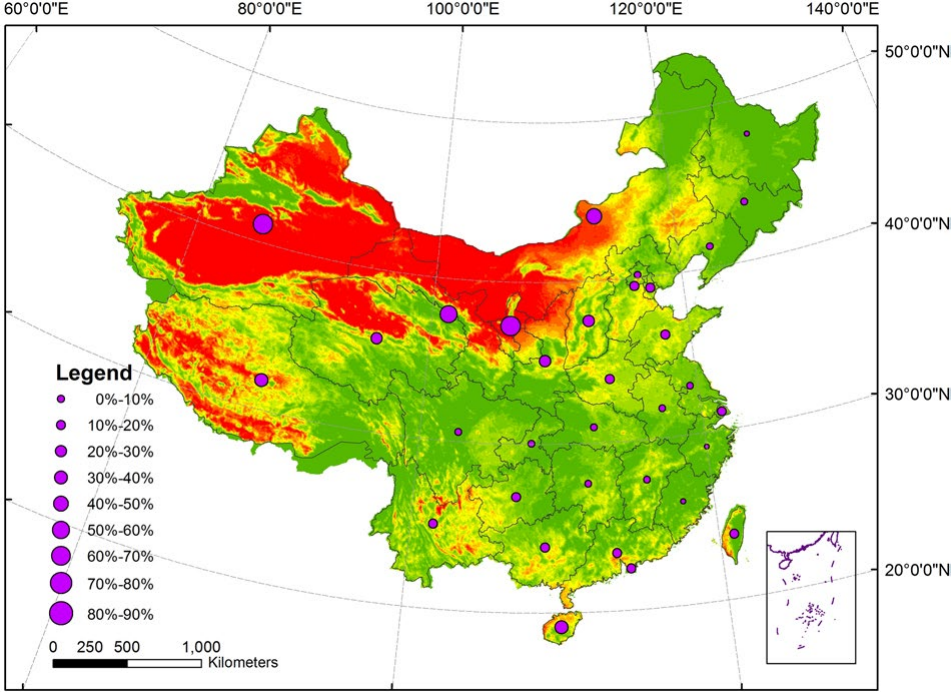
Mean drought frequency in China’s provinces, municipalities and autonomous regions from 1982 to 2010. The map was created using ArcGIS 10.2 (ESRI Inc., Redlands, California, USA).

There is also clearly difference in mean drought frequency between provinces, municipalities and autonomous regions in the past 30 years (Fig. 3). High frequency droughts (>50%) appeared in Ningxia, Xinjiang, Gansu and Inner Mongolia. The mean drought frequencies in Shaanxi, Shanxi, Qinghai, Shandong and Tibet were between 30% and 50%. The mean drought frequency in Heilongjiang, Zhejiang and Fujian were much lower (<10%).

### Spatial distribution of frequency of different level drought based on mTVDI in China from 1982 to 2010

**Figure 4:**
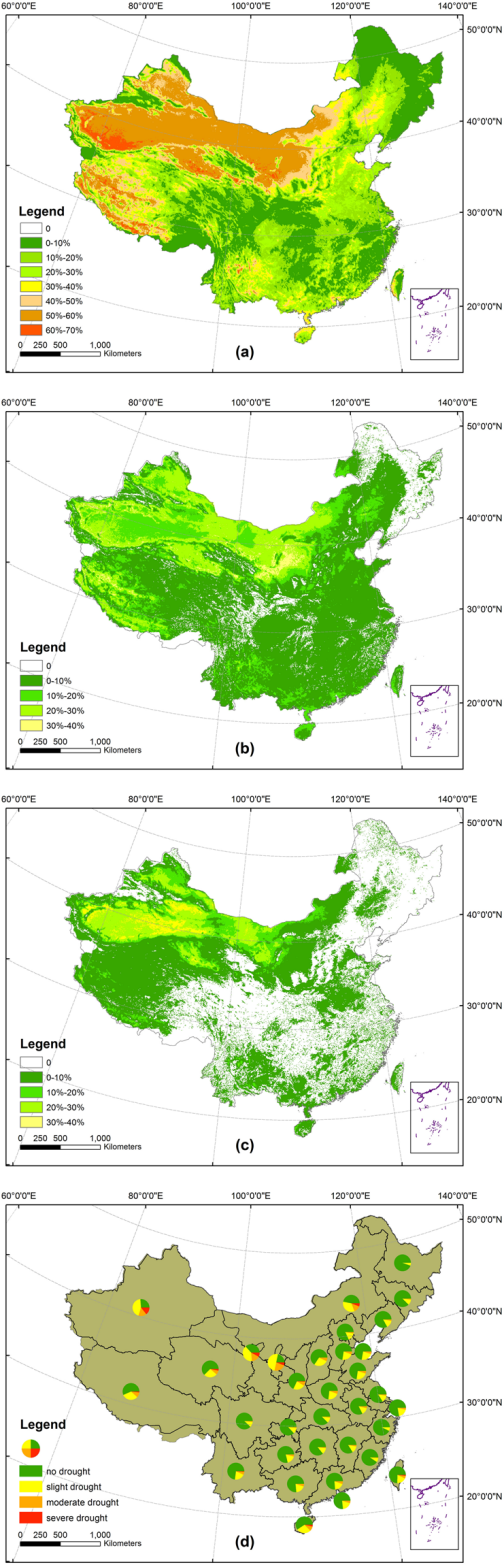
Spatial distribution of frequency of (**a**) slight drought, (**b**) moderate drought, and (**c**) severe drought in China and (**d**) in provinces, municipalities and autonomous regions from1982 to 2010. The map was created using ArcGIS 10.2 (ESRI Inc., Redlands, California, USA).

The frequencies of different levels of droughts (slight, moderate and severe) were counted in the national (Fig. 4a,b and c) and provincial levels (Fig. 4d). The frequencies of slight drought were higher than those of moderate drought and severe drought while the frequencies of moderate drought were higher than those of severe drought (Fig. 4a,b and c). In the provincial level, the proportions of slight drought were higher than those of moderate drought, and the proportions of severe drought were much lower (<15%) (Fig. 4d). Gansu, Ningxia, Xinxiang, Inner Mongolia and Qinghai province were all prone to severe drought with the mean frequency higher than 5%, and the highest frequencies of severe drought appeared in Gansu. Moderate drought often occurred in Ningxia, Xinxiang, Gansu, Inner Mongolia, Shaanxi, and Qinghai with the mean frequency higher than 7%.

### Temporal variation of drought based on mTVDI in China from 1982 to 2010

**Figure 5:**
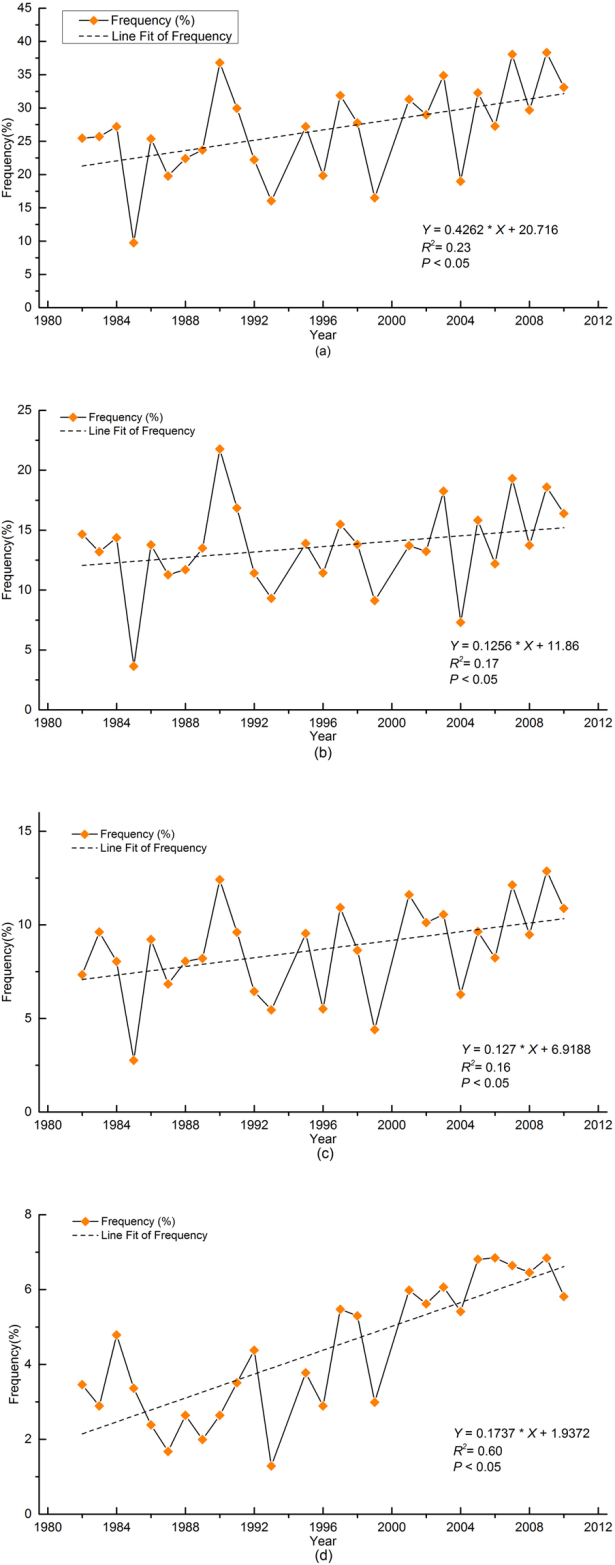
Annual average frequency of (**a**) all droughts, (**b**) slight drought (**c**), moderate drought and (**d**) severe drought in China from 1982 to 2010.

In order to analyze the temporal pattern of droughts in China during the past decades, the number of occurrence of all droughts and different levels of droughts (slight, moderate and severe droughts) were calculated annually. The annual occurrence frequencies of drought fluctuated between 10% and 40% during the research period (Fig. 5a). In particular, the occurrence frequencies of drought in 1985–1986, 1990–1991, 1999–2001, showed big changes. The annual frequencies of drought after 2001 still fluctuated, but the magnitude was much smaller than those before 2001. Despite fluctuation of occurrence frequencies of drought, there was a clear increasing trend during the last decades.

### Temporal variation of different levels of drought based on mTVDI in China from 1982 to 2010

The occurrence frequencies of slight drought fluctuated between 3% and 25% (Fig. 5b). The annual frequencies of moderate drought fluctuated between 3% and 13% (Fig. 5c). The annual frequencies of severe drought fluctuated between 2.89% and 6.84%, smaller than those of slight and moderate drought (Fig. 5d). There were increasing trends of occurrence frequencies of different levels of drought in the period 1982–2010, while the rising trend of severe drought is much clear than those of slight and moderate droughts (Fig. 5b,c and d). In particular, frequencies of severe droughts increased rapidly from 1999 to 2010 (Fig. 5d).

**Figure 6:**
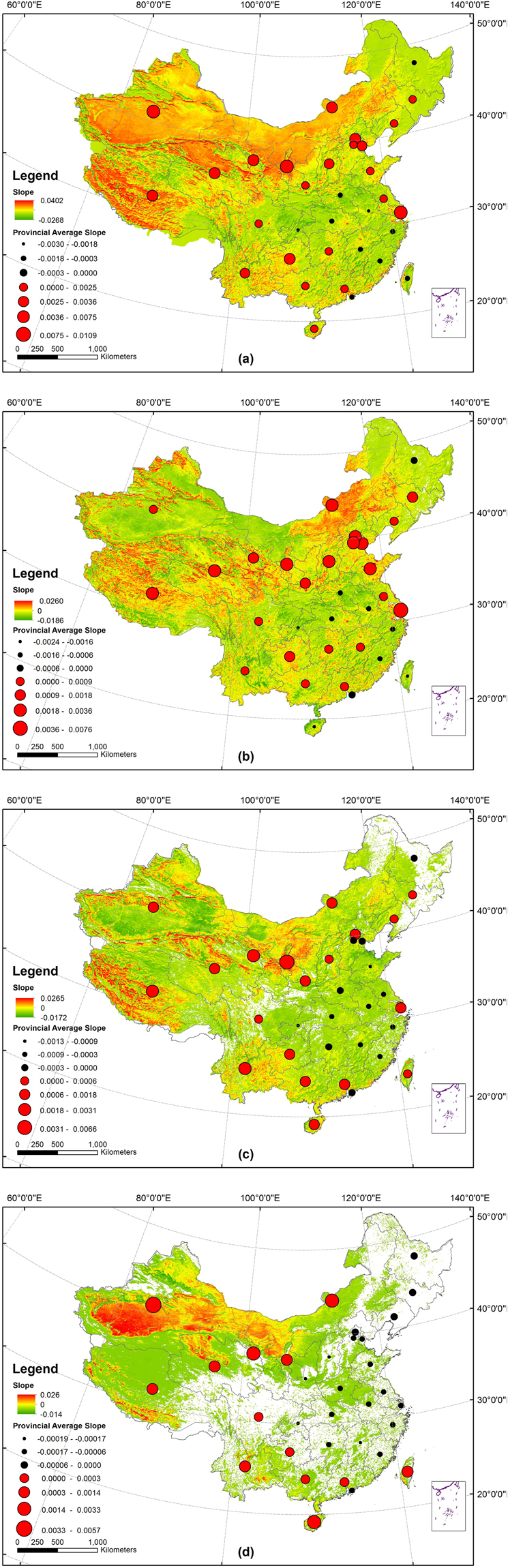
The average slopes of (**a**) all droughts, (**b**) slight droughts, (**c**) moderate droughts, and (**d**) severe droughts, in China’s provinces, municipalities and autonomous regions from 1982 to 2010. The map was created using ArcGIS 10.2 (ESRI Inc., Redlands, California, USA).

In order to analyze the change pattern of droughts, increase or decrease, the tendency rate of drought frequency (see Material and Methods) was calculated based on the mTVDI. The average slopes of drought frequency in China’s 34 provinces, municipalities and autonomous regions were calculated (Fig. 6a). The slopes of drought frequency in 23 provinces, municipalities and autonomous regions were more than 0 during the past decades, indicating the rising frequencies of droughts in these areas. In particular, the slopes of drought frequency in Ningxia, Gansu, Xinjiang and Shanghai were more than 0.006, indicating remarkable increasing trends of drought. The slopes in Sichuan, Liaoning, Hunan and Jiangsu provinces were close to 0, indicating the relatively stable frequencies of drought. The slopes of drought frequency in 11 provinces, municipalities, and autonomous regions were less than 0 (Fig. 6a), indicating the decreasing frequencies of drought.

The slopes of three levels of drought were also calculated (Fig. 6b–d). Slopes of the slight drought frequency in 23 provinces, municipalities and autonomous regions were greater than or equal to 0, while those in 11 provinces, municipalities and autonomous regions were less than 0 (Fig. 6b). In particular, the slopes of slight drought frequency in Qinghai, Ningxia, Shandong, Shanghai, and Tianjin were more than 0.002.

The slopes of moderate drought in 20 provinces, municipals and autonomous regions were greater than 0 (Fig. 6c). In particular, the slopes of moderate drought in Ningxia, Tibet, Gansu and Yunnan were greater than 0.002. The slopes of moderate drought in 14 provinces, municipals and autonomous regions were less than or equal to 0, especially, Chongqing, Jiangxi less than 0.

The slopes of severe drought frequency in China were distributed unevenly (Fig. 6d). The slopes in Gansu, Hainan, Inner Mongolia, Ningxia, Qinghai and Xinjiang were more than 0.0012, much bigger than other areas. Slopes of drought frequency in 22 provinces, municipals and autonomous regions fluctuated between −0.0005 and 0.0005, indicating the slight changes of severe drought in these areas. In conclusion, the overall frequencies of severe drought in the whole country showed an increasing trend.

## Discussion

In countries with large area, the altitude difference can be huge. In particular, the altitude difference between eastern and western China is more than 4000 m. However, TVDI ignored the effect of elevation on temperature, which reduces the accuracy of temperature remote sensing product in high latitude areas^36^. In addition, as a result that vegetation possesses the trait of self-protection, rising temperature does not necessarily mean that vegetation must suffer from lack of water. Therefore, the fitting dry edge cannot really match the real dry edge, indicating the excessive detection for drought based on TVDI^37^. Different from many previous studies, this paper integrated elevation correction and dry edge correction for TVDI and proposed a new drought index mTVDI based on the 0.05° remote sensing data. To validate the sensitivity of mTVDI to soil moisture, the *in situ* soil moisture data was utilized. The results showed that the mTVDI was more suitable to monitor drought in a large scale region than TVDI, with its highest correlation coefficient and relatively smaller Bias, RMSE and MAE (Table 1). In general, TCI monitors drought following temperature only while AVI and VCI consider both temperature and NDVI^17–19^. TVDI, which considers the relationship between temperature and NDVI^32^, has clearer physical meaning and more exact precision for drought monitoring. Given the limitation of TVDI when used for drought monitoring in a large scale with great elevation variation, mTVDI combines elevation correction and dry edge correction for TVDI, thereby leading to a best performance among the drought indices (Fig. 1 and Table 1).

The spatial distribution and temporal variation of drought frequency from 1982 to 2010 in China was estimated based on mTVDI in this paper. Droughts appeared frequently in Northwest China and the southwest of Tibet while drought centers of North and Southwest China appeared in Huanghuaihai Plain and Yunnan-Guizhou Plateau, respectively. The frequency of drought increased as a whole while the frequency of severe drought increased markedly by 4.86% and the frequency of slight drought increased slowly by 3.56% during the period of 1982–2010.

Some researchers have focused on the spatial-temporal distribution of drought in China based on the gauge station data or gridded data of lower spatial resolution^38,39^. For examples, based on the Standardized Precipitation Index (SPI), Standardized Precipitation Evapotranspiration Index (SPEI) and China Integrated Drought Monitor (CI) calculated by gauge station data, Ayantobo *et al*. found that severe drought occurred more frequently in North China and Northwest China from 1961 to 2013^38^. Based on the 0.25° gridded indices of SPI, RDI and SPEI, Xu *et al*. found that the western part of North China Plain, Loess Plateau, Sichuan Basin and Yunnan-Guizhou Plateau had a marked trend of drying from 1961 to 2012^39^. In spite of different methods used in these studies, the general patterns of these studies are good accord with our results in Fig. 2. Many papers above have focused on the drought development in China in a long time series. However, there are comparability in the temporal-spatial distribution and change trend of drought in China between this study and previous studies^38,39^. It is worth noting that the gauge station data used in above studies are too sparse to be used in such a large scale of China, and the data of 0.25° spatial resolution also is less sufficient to monitor drought in detail compared with higher spatial resolution data^40,41^. In this paper, the frequencies of different levels of droughts (slight, moderate and severe) were analyzed in provincial levels in China (Fig. 4d).

There were also some studies investigating the spatiotemporal patterns of drought in China based on the comprehensive drought monitoring method^42,43^. Using Integrated Surface Drought Index (ISDI), Zhou *et al*. found that the areas with the most obvious decreasing trend appeared in Northeast China and the south of the Yangtze River from 2001 to 2013^44^, which is similar to our results shown in Fig. 6(a). However, these papers covered a shorter period of the spatiotemporal patterns of drought in China, only around a decade.

Different from previous studies, there are great differences in the distribution of drought frequency in Xinjiang and Tibet^38,39,44^ due to the proposed index mTVDI in this paper, which considers the great elevation variation of China and the correction for dry edge.

Our study made a great improvement of drought monitoring index, and the results of spatial-temporal distribution of drought based on mTVDI could provide references for China’s drought development and extreme weather which are useful for the policy making of climate change. This paper considers elevation and dry edge factors, but the accuracy of TVDI is still affected by longitude, latitude and other factors for large scale drought monitoring^23,43^. In further studies we will take consideration of longitude and latitude so that it can further improve the accuracy of TVDI for national drought monitoring.

## Material and Methods

### Datasets

The remote sensing data utilized in the current research are MODIS level 3 product data, named MOD13C2 and MOD11C3, and the reflectance data AVHRR09C1 obtained from NASA’s Earth Observing System Data and Information System (http://reverb.echo.nasa.gov/).

**Figure 7:**
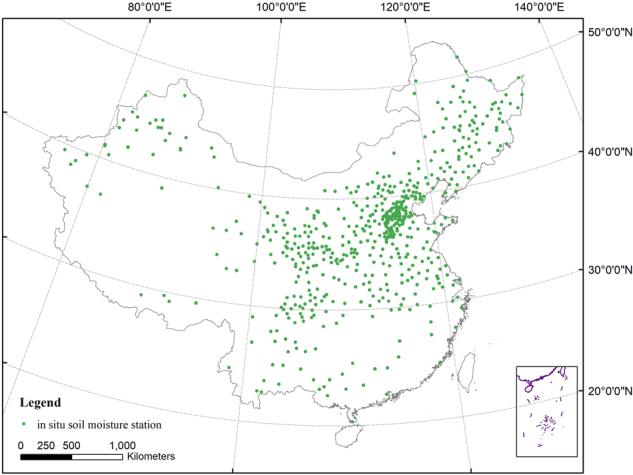
The distribution of the *in-situ* soil moisture stations used in this paper. The map was created using ArcGIS 10.2 (ESRI Inc., Redlands, California, USA).

*In situ* soil moisture data were collected from the data set of China Soil Moisture on the Growth and Development of Crops in China (http://data.cma.cn/site/index.html) provided by China Meteorological Science Data service network. The distribution of the *in situ* soil moisture stations was shown in Fig. 7. Four months (March, May, July and November) for each season in 2001, 2003, 2005, 2007, and 2009 were chosen to validate the reliability of the drought index mTVDI.

Digital Elevation Model (DEM) data were obtained from Shuttle Radar Topography Mission (SRTM) V4 (Geo-tiff format) and International scientific data mirror service system (https://www.usgs.gov/). The spatial resolution is 90 m. After data resampling, the spatial resolution of the DEM data reached 0.05°.

### Calculation of *T*_*s*_ and NDVI

The synthesis of temperature products were calculated using the following formula^45^:

2 $${T}_{s}=[{T}_{4}+3.33\ast ({T}_{4}-{T}_{5})]\ast 0.99+0.0075\ast {T}_{5}$$

where ${T}_{s}$ represents the surface temperature; ${T}_{4}$ and ${T}_{5}$ represent AVHRR09C1 data in the band 4 and 5, respectively. NDVI were calculated as follows^24^:

3 $$NDVI=\frac{({B}_{2}-{B}_{1})}{({B}_{2}+{B}_{1})}$$

where *B*_1_ and *B*_2_ represent the red band and near-infrared band, respectively. According to the Eq. (3), the surface temperature and NDVI from 1982–2010 were calculated.

### Calculation of Temperature Vegetation Dryness Index (TVDI)

If study area contains different kinds of land cover, from water to sparse vegetation and dense vegetation, the scatter diagram of surface temperature and vegetation index usually presents as in triangulars^24^ or trapezoids^39^. Based on the NDVI-Ts space, Sandholt^28^ developed a temperature vegetation drought index (TVDI). TVDI were calculated as follows:

4 $${\rm{TVDI}}=\frac{{T}_{s}-{T}_{smin}}{{T}_{smax}-{T}_{smin}}$$

where *T*_*s*_ represents the surface temperature, *T*_*smax*_ represents the highest surface temperature corresponding to *NDVI*, and *T*_*smin*_ represents the lowest surface temperature corresponding to *NDVI*. The calculations of *T*_*smax*_ and *T*_*smin*_ are as follows.

5 $${T}_{smax}={a}_{1}+{b}_{1}\ast NDVI$$

6 $${T}_{smin}={a}_{2}+{b}_{2}\ast NDVI$$

### Calculation of the modified Temperature Vegetation Dryness Index (mTVDI)

#### Elevation correction for the surface temperature

Temperature declines 0.6 °C when height rises 100 m^46^. The altitude difference between eastern and western China is more than 4000 m. However, TVDI doesn’t consider the effect of elevation on temperature and this can reduce the accuracy of drought estimate in high latitude areas. Thus, before calculating the vegetation index, the temperature data need to be corrected according to the elevation as follows:

7 $$T(H)=T+a\ast H$$

where *T*(*H*) is the corrected temperature; *T* is the temperature before correction; *H* is the elevation of calibration points; and *a* is the elevation correction coefficient (0.006).

#### Dry edge correction

Vegetation possesses the trait of self-protection. When the temperature reaches the threshold value, vegetation will close their stomas to decline transpiration^37^. Therefore, rising temperature doesn’t mean that vegetation must suffer from lack of water and eventually the fitting dry edge cannot really match the real dry edge, indicating the excessive detection for drought based on TVDI^37^. Due to the non-linear relationship between the fitting dry edge and soil moisture, the correction value in dry edge and moisture is also non-linear^37^. Stisen *et al*. utilized the relationship between vegetation index and evapotranspiration to derivate the relationship between the correction value in dry edge fitting and vegetation index^47^. The dry edge was corrected as follows:

8 $${\rm{C}}={\rm{\alpha }}{(\frac{NDV{I}_{i}-NDV{I}_{{\rm{\min }}}}{NDV{I}_{{\rm{\max }}}-NDV{I}_{{\rm{\min }}}})}^{2}$$

where *C* represents correction value; ${\rm{\alpha }}$ represents the correction coefficient, *NDVI*_*max*_ and *NDVI*_*min*_ represent the max and the minimum *NDVI* respectively.

#### mTVDI

After the correction of elevation and dry edge, mTVDI were calculated as follows:

9 $$\begin{array}{c}mTVDI=\frac{T(H)-{T}_{smin}}{{T}_{smax}-{T}_{smin}}-C\\ =\,\frac{T+a\ast H-{T}_{smin}}{{T}_{smax}-{T}_{smin}}-\alpha {(\frac{NDV{I}_{i}-NDV{I}_{min}}{NDV{I}_{max}-NDV{I}_{min}})}^{2}\end{array}$$

where *T* represents the uncorrected temperature; *H* represents the elevation of correction points; $a$ represents the coefficient of elevation correction; *T*_*smax*_ represents the max surface temperature corresponding to *NDVI*; *T*_*smin*_ represents the minimum surface temperature corresponding to *NDVI*; *T*_*smax*_ and *T*_*smin*_ can be calculated by formula (5) and (6). *NDVI*_*max*_ and *NDVI*_*min*_ indicate the max and minimum *NDVI* respectively; ${\rm{\alpha }}$ indicates the coefficient of dry edge, which implies the influence of the minimum evapotranspiration on dry edge.

### The calculation of the tendency rate of drought

In order to analyze the change pattern of droughts, increase or decrease, the tendency rates were calculated based on the mTVDI as follows^48^:

10 $$slope=\frac{n\times {\sum }_{i=1}^{n}(i\times {P}_{i})-({\sum }_{i=1}^{n}i)\times ({\sum }_{i=1}^{n}{P}_{i})}{n\times {\sum }_{i=1}^{n}{i}^{2}-{({\sum }_{i=1}^{n}i)}^{2}}$$

where *i* is the series number of the year; *n* is the length of time series; *P*_*i*_ represents the frequency of drought occurring in the *i*_*th*_ year. If the slope is positive, it represents that the occurrence frequencies of drought increase, and the degrees of drought worsen during the research period. On the contrary, the negative slope indicates degrees of drought alleviate.

### Map making and data statistical analyses

All maps were created using ArcGIS 10.2 (ESRI Inc., Redlands, California, USA). All statistical analyses were conducted using SPSS 22.0 (IBM Inc., Armonk, New York, USA).
